# New Jersy State Society

**Published:** 1875-10

**Authors:** 


### NEW JERSEY STATE SOCIETY.

The N. J. State Dental Society held its fifth annual session at Long Branch, July 6th, 7th and 8th. President Brown, of Mount Holly, presiding the first day occupied was with the election of the following new members: Drs. L. S. Marsh, H. S. Parke, D. H. Thickstun, Wm. E. Frances, Chas. W. Meloney, W. W. Dooland and C. M. Merritt. The Board of Examiners report that during the year past they had examined and gave certificates to practice to the following dentists: Drs. Wm. E. France, F. J. Leonard, A. L. Strecher and R. J. Reed. The session of Wednesday opened with the President's address from which we make the following extracts: Giving an interesting resume of the year, he spoke of amalgams as having renewed attention given to its use by careful tests and experiments, that amalgam properly prepared and used will preserve a tooth for years, is beyond a doubt, improperly used is worse than useless. Celluloid has taken great strides the past year in public favor, and is fast superseding the use of rubber. Beautiful in color, strong in texture, easy of manipulation, it bids fair to take the place of all other materials. As a base for artificial teeth, it appears to be just what was wanted to relieve us from the obnoxious Rubber Company.

I think every dentist who has learned to use it will never go back to rubber. Our law regulating the practice of dentistry is working well, though being much more stringent than the N. Y. Law, yet no fault has been found in its workings. The urging upon the society of prohibiting any member receiving a student for a less period than three years and making it obligatory upon the student to attend two courses of lectures and graduate at some dental college, and I would advise the passage of a resolution that after three years, no one should be received as a member who had not complied with the same. The address was well received and elicited considerable discussion. The 1st essay by Dr. T. B. Welch, of Vineland, was then read and well received. Extracts, as follows: Some hurry through their dental operations from selfish motives, they are in haste to be rich and therefore anxious to make every hour of labor count the greatest number of dollars, nearly all such come to grief as they should, some who are naturally nervous and excitable are not aware of a want of care it being a misfortune and the sooner they overcome this weakness the better workman they will be; others hurry through from a desire to be considered fast workers, continually boasting of how many teeth they can fill in an hour and how many teeth they can extract in a minute, this is foolish, we should work as rapidly as we can consistently with accuracy, the greatest pride of the true workman is in the perfection of his work and he is generally modest in his claim. Let our reputations speak of honor, dignity and success. Dr. C. S. Stockton of Newark followed with an Essay on Dental Education of which the following is a synopsis, the 1st point was the extreme haste of the Profession in manufacturing Dentists of their students.

The early and thorough education of him who proposes to enter the Profession of Dentistry was demanded; the eagerness of the American People to enter business life and the haste to get rich are the glory and vice of America.

The uneducated man and dentist go out in the world and are done. Letters, Philosophy, and the Sciences have no interest to him and to their delights he will forever remain closed. And he claims that he is not appreciated at his true worth it is his own fault for if he enter the circle of the educated he must educate himself to come to their standard-there are so many men not blessed with the best qualifications and the number has been so increased that the time has come that societies like this should make their voices heard. I would have no one commence the practice of dentistry at this day until he is a graduate of a Dental College unless he has spent two years in the office of a competent Practitioner.

Colleges are blamed in a manner for the way they make Dentists, in a measure they are unjustly censured; the colleges have done more for the profession than all other means combined. Young men are taken by some of our Practitioners sent at once to college without any previous instruction and the college in eight months is expected to make thorough Dentists of them. Let us cherish our State Society as I know of no better way of promoting dental education in N. J. than by faithful attendence upon its sessions, what possible ambition to high deeds can a man have who lives wholly within himself, let us then as our means to higher professional attainments cherish this association educate each other and the Dentists of N. J.

Dr. Welch agreed with Dr. Stockton's citing instances that we all require a differance in time to gain the practical and theoretical education necessary to become skillful practitioners.

Dr. Hayhurst considered all general laws bore hard in certain cases, but better it be so than to open the flood gates to incompetency.

Dr. Kingsley thought the pupil should study 3 years and pass through college as the paper recommends. Dr. J. R, Cobb differed entirely and thought it should not be obligatory upon students to attend dental cclleges and spoke of the incompetency of graduates for every day practicla work upon leaving college they wishing to perform all operations in the manner and way they did in college. He denounced the system of selling diplomas, having proof of a certain Philadelphia college granting a diploma which the N. J. Board of Censors refused to grant because the candidate was wholly incompetent, the diploma of the college, in his estimation, is no guarantee a fitness to practice.

Dr. Hanks defended the college system but condemned their abuses and thought the present system of dental and medical colleges wrong. Dr. Hayhurst, Ex-college professor, cited the thoroughness of the examination and did not consider that the Board of Censors had as good an opportunity and the time at command as a college faculty.

Dr. C. A. Meeker recommends that the examinations of the Board of Censors be so thorough that the diploma of the Society will be an honor to the holder, though the candidate holds a degree of D. D. S., also as Dr. Stoekton says, to work for the elevation, education and character of the Society. After considerable discussion by others the subject was closed.

Dr. Geo. H. Perine, of New York, by special request, spoke at some length upon the use of the Galvanic Cautery, for oral surgery; Dr. Perine said there was advance in all departments of science, particulary so in our own department, dental surgery. He claims that the introduction of the cautery, (he being the first person who has applied that particular cautery in oral surgery,) is the advance of the age, and he strongly recommended its uses, if the cautery be employed for obtunding sensitive dentine he would advise its application with great care and only in cases where the instrument can be applied directly to the tooth to be operated upon, guarding the instrument from contact with any other tooth, the action will be instantaneous and effectual. Dr. Perine illustrated the use of the battery for operations in oral surgery and presented the instrument he uses. The advantages he set forth are, that the operation is instantaneous painless and without shock to the body which it does not in any degree effect unpleasantly, the application is easy, free from hemorrhage, (which is of great moment in surgical operations in the mouth) and finally the reparative process is rapid.

Next followed Dr. J. R. Goble, of Hoboken, who read a highly interesting paper on the cause and cure of the absorption of the alveolar process taking the ground that local irritation was the primary cause preceded by salivary calcus, diseased teeth, etc. The cure of which was to remove the irritation with delicate instruments, keeping the necks free and well polished, and recommending the use of a weak solution of chlorate of potash. He deprecated the custom of so much scrubbing the teeth, citing numerous instances of absorption and denuding of the enamel.

Prof. Abbott, of New York, said he was well pleased with Dr. Goble's remarks, and agreed with the doctor in every particular and hoped all dentists would instruct their patients in the use of the tooth brush, he also by special request, spoke of the use of Salicylic Acid for use in disease of the mouth, it having strong antiseptic properties and withal being perfectly harmless. Dr. Kingsley and others recommended the use of Aconite and Iodine in saturated solution for inflammation and for use after filling nerve cavities. On motion, the President was authorized to welcome to N. J., the American Dental Convention which meets at Long Branch in August. Adjourned.

MORNING SESSION, JULY 8th.

The Invention of Dr. Chevalier in regard to an improvement in metallic bases was delegated to a committee of Drs. Stockton, Meeker and Pimey, to report at next meeting.

The Dental Luminary, a small pamphlet designed for distribution by dentists among their patients, which would enable the reader to discriminate a good from an inferior dentist. The committee spoke very highly of it and it was ordered printed by the Society provided enough copies by individual members would be ordered to make it practicable. Atlantic city was decided upon as the next place of meeting. Drs. Goble, Stockton, Welch and Hayhurst were elected delegates to the N. Y. S. D. S.

Dr. J. W. Hayhurst, of Lambertville, next spoke on the best method of inserting partial sets of teeth. In taking the impression plaster was considered the best article in use. The contact by the plate on the natural teeth was by him not considered necessary, the atmospheric pressure being sufficient when the impression was perfect, every thing depended on making a perfect fit. Dr. Dibble thought an air chamber not necessary, made all his plates without them Dr. F. W. Barbour gave his method in using wax and plaster in difficult under cuts. Dr. Meeker asked essayist the best place to put the air chamber, the doctor thought in a high roof, near the end of the plate, and in a flat roof, near the centre, deprecated the custom of having so many angles to them. Dr. Stockton considered gold the best for partial sets and continuous gum for full sets. As the subject had received considerable attention and latitude the debate was closed and Dr. E. F. Hanks invited to read his essay on Amalgam.

After Dr. Bogue's able and exceedingly thorough exposition of amalgams under every condition that we may be called upon to use them, it seems to me presumption to say anything on the subject unless one can say something new. But I have the consolation that I am not alone in this, for so long as dental societies exist, just so long will we be called upon to give a re-hash of what has been said and done before.

When I took the subject, I intended to go much more fully into this matter than I have until I saw Dr. Bogue's article which covered the ground so completely and much more scientifically than I ever hoped to do, that I have contented myself with making only a few experiments.

I would suggest before all things that we be candid in what we say in the discussion that may follow, and say exactly what we mean and not exactly what we do not mean on this important subject. Time and time again have leading men in the profession denounced before dental societies the use of amalgam in any manner or under any circumstances, returning home to use it privately in their daily practice. Amalgam has simply been unfashionable, and the young men with reputations to make have been warned that they could not afford to speak favorably of it. Now, all that is changed. Amalgam has become the rage. The journals as well as the teeth are filled with it, and voluminous papers are read before learned societies about it.

While amalgam was under the ban tons of it were made, but no one used it. The manufacturers said they sold it to the dental depots: the depots said the dentists bought it; but the dentists said they never used it. [Laughter.] If this was true, what a tremendous stock some of us must have, now that amalgam has grown to be so popular! But now that

Fletcher, Bogue, Hitchcock, Cutler and others have taken up the subject and proved that amalgam is not the bugbear we have been taught to believe it was, I fear that foolish and unskillful men will now push the use of it to extremes, as they have rubber, heavy foil, and a great many other things that are very useful in their proper places.

Amalgam is no better nor worse to-day than it has been for years, with perhaps a few exceptions, and in my opinion it should retain the same place it has always held in the hands of good operators—that is a cheap substitute for gold in posterior fillings, as rubber and celluloid are cheap substitutes for gold and platinum for artificial dentures, only to be used when the patient is too poor or too mean to pay for the better article.

I would also suggest amalgam for badly decayed teeth, where the expense would be great and the success of the operation doubtful.

The object of most writers has been, I think, to test by actual experiment the theories and assertions advanced from time to time on this subject, and to find out the exact scientific basis that amalgam has to rest its claims upon as a useful agent in our daily practice, and not to make converts to the use of amalgam as some unreasonably think.

The following is a specimen of the groundless assertions that have been recklesly made from time to time.

Dr. Payne in the Chicago Medical Journal speaks of the poisoning of thousands of people all over the world from corrosive sublimate generated in the mouth from amalgam fillings.

This assertion is pretty effectually answered by the certificate of analysis of Prof. Chandler, of Columbia College addressed to Dr. Bogue.

"Sir: The samples of saliva in which various alloys (amalgams) had been digested, submitted to me for examination contain no mercury in solution."

These samples of amalgam, with the teeth in which they were placed, before being submitted to Prof. Chandler, had been immersed for three months in various acids so powerful as to nearly dissolve the teeth.

There are three samples of each of the following amalgams before you: Arrington's, Townsend's, Lawrence's, "Extra," Fletcher's and Kearney's. I do not see any great difference in the working of these amalgams, with the exception of Extra amalgam which works poorly.

In conclusion I would say if almost any amalgam is used with care, teeth can be filled to preserve them without injury to the health; but as for putting it on a par with gold I certainly do not except the few cases already mentioned.

The paper was generally discussed and the remarks coincided with the views of nearly all present.

The last essay by Dr. De Lange was of importance to the profession generally, and was awarded a vote of thanks. It was entitled Celluloid *vs.* Rubber, and was as follows:

Bad are the goods when two masters chase one man. Good for the man. Two men have two workmen. Good for the consumer. So it is with Rubber and Celluloid.

Before 1870 we used but one material, we employed but one workman; since we have had others toilers, and ergo, other substances; for, if "necessity is the mother of invention," competition is certainly its father.

It is due to the earnest efforts of the Messrs. Hyatt. that Celluloid has reached even its present state; as the compound we have now, certainly has more plasticity, and less inflammability than the collodion obtained by the process first patented.

In experimenting with camphor and soluble cotton, they were mixed and casually squeezed in the hand—when it was found that they compacted and showed signs of combination. It was previously known that a solution of camphor in alcohol was a solvent of the soluble cotton, but this proved that alcohol need not be used; and, that camphor was, under proper conditions a perfect solvent.

It was then ascertained that a chemical combination took place; and a short time after the first experiment, by heating the material to a lower temperature than necessary to melt camphor, a lump of solid celluloid was produced ; subsequent experiments have developed the fact that a smaller quantity of camphor could be used, thereby making better plates, and almost doing away with the unpleasant recollections of last winter's clothing.

One of the greatest objections against celluloid, formerly urged by the vulcanists was, as we have said before, the unpleasant camphor taste, and we beleive this is still put forward by some of the non-experimenters. This difficulty has been well nigh, if not entirely, overcome within the last year or two.

In working this substance no disagreeable odor is noticed, as there doubtless is in rubber; in fact this is quite a point against rubber, for in manufacturing it, sulphur is used, and we all know what a delightful perfume results from the combined effects of escaping sulphurous gas and heated eaoutchouc. Not only is the escaping gas offensive, but it is unhealthy; a fact which could never have been stated of celluloid, even when in its condition of greatest imperfection; the odor of camphor, though not pleasant to all, is beyond denial unhealthy to none, and even this is passing from us. The rubber retains its obnoxious scent even after completion.

That the new compound is more agreeable to the mouth, is easily judged from the remarks of the patients, and the general satisfaction that is given.

It is much lighter than rubber, and decidedly more susceptible to a high polish when we have the set complete; and together with these cleanest properties it also refrains from heating the mouth as the old material does.

The whole substance has now properties of lightness, toughness and strength, together with a resisting power, which vulcanized goods never had, or never will possess.

Celluloid now is entirely different from that which was first manufactured. Dentists who made use of it at that time had obstacles which they thought they would never surmount. These failures induced a number of the experimenters to toss their celluloid, and apparatus, to the waste pile, and return to their first love—Uncle Josie.

We quote from the "Cosmos" of March, 1872, the remarks of Dr. Earnes, as follows:

"Sufficient time has now elapsed to prove, we think, the utter worthlessness of this material as a base for artificial teeth, and we publish the following list of experimental cases with this base, with the results, as our own experience, and upon which, in part, we have our opinion of its merits.

After which follows a long and most melancholy list of failures, both in color and texture. His plates ran from pink to dark brown and then warped and shrunk to such a degree that sometimes the plate would not touch the center of the arch by one-fourth of one inch. It appeared that all of his cases were returned in about three weeks or less. Unfortunate man.

No dentist ever thinks of the failures made and the trouble caused by rubber on its first introduction? I do, for at that time I acted as an agent for the Goodyear Rubber Co. and had more, much more difficulties in introducing it than the agents for the celluloid have had in bringing their base before the public.

Dentists howled at the idea of using rubber in the mouth: they said it was unclean, that it smelled badly, and I well remember several prominent members of the profession, among whom was Dr. Elisha Townsend, of Philadelphia, (now deceased.) stating that he would not have anything to do with a material injurious to the mouth.

The feeling was so cogent that some dentists advertised that the new base would not be used in their establishment, but they did use it evidently, as it was the only means of self protection, and with the exception of the metals, it held undisputed sway for a period of more than fifteen years.

But now another base has interfered with the even tenor or its ways, and in celluloid it will meet its Brutus.

Does it not argue strongly for the excellence of the new material, or the perniciousness ot the old, that this composition should, in less than five years stand the mighty rival of rubber, with its twenty years of experience, and "millions in it."

